# Potassium ion channels as a molecular target to reduce virus infection and mortality of honey bee colonies

**DOI:** 10.1186/s12985-023-02104-0

**Published:** 2023-06-22

**Authors:** Christopher J. Fellows, Michael Simone-Finstrom, Troy D. Anderson, Daniel R. Swale

**Affiliations:** 1 Department of Entomology, Louisiana State University AgCenter, Baton Rouge, LA 70803 USA; 2 USDA-ARS Honey Bee Breeding, Genetics, and Physiology Laboratory, Baton Rouge, LA 70820 USA; 3 Department of Entomology, University of Nebraska, Lincoln, NE 68583 USA; 4 Department of Entomology and Nematology, Emerging Pathogens Institute, University of Florida, 2055 Mowry Road, PO Box 100009, Gainesville, FL 32610 USA

**Keywords:** Kir, Honey bee, Reactive oxygen species, IAPV, Honey bee virus, Antiviral immunity

## Abstract

Declines in managed honey bee populations are multifactorial but closely associated with reduced virus immunocompetence and thus, mechanisms to enhance immune function are likely to reduce viral infection rates and increase colony viability. However, gaps in knowledge regarding physiological mechanisms or ‘druggable’ target sites to enhance bee immunocompetence has prevented therapeutics development to reduce virus infection. Our data bridge this knowledge gap by identifying ATP-sensitive inward rectifier potassium (K_ATP_) channels as a pharmacologically tractable target for reducing virus-mediated mortality and viral replication in bees, as well as increasing an aspect of colony-level immunity. Bees infected with Israeli acute paralysis virus and provided K_ATP_ channel activators had similar mortality rates as uninfected bees. Furthermore, we show that generation of reactive oxygen species (ROS) and regulation of ROS concentrations through pharmacological activation of K_ATP_ channels can stimulate antiviral responses, highlighting a functional framework for physiological regulation of the bee immune system. Next, we tested the influence of pharmacological activation of K_ATP_ channels on infection of 6 viruses at the colony level in the field. Data strongly support that K_ATP_ channels are a field-relevant target site as colonies treated with pinacidil, a K_ATP_ channel activator, had reduced titers of seven bee-relevant viruses by up to 75-fold and reduced them to levels comparable to non-inoculated colonies. Together, these data indicate a functional linkage between K_ATP_ channels, ROS, and antiviral defense mechanisms in bees and define a toxicologically relevant pathway that can be used for novel therapeutics development to enhance bee health and colony sustainability in the field.

**Supplementary Information:**

The online version contains supplementary material available at 10.1186/s12985-023-02104-0.

## Introduction

The western honey bee, *Apis mellifera* L., is the most widely managed crop pollinator and significantly contributes to the economic viability and sustainability of the food and fiber industry. Declines in the numbers of both managed and wild pollinators have prompted researchers to intensify efforts to understand the factors driving these declines [1–3]. While a variety of factors negatively impact bee populations, virus infections are significant drivers of colony losses and, hence, viruses are considered a major threat to the health of bees at the individual and colony levels [4–7]. Managed bee colonies are commonly infected with multiple viruses concurrently and, although such infections may remain asymptomatic in healthy colonies, exposure to environmental stressors can weaken colony immunity and provoke acute virus outbreaks that can lead to bee death and colony loss [8, 9]. Further, infection with Deformed Wing virus (DWV) leads to immunosuppression in the bee and has been suggested to lead to greater reproduction of the *Varroa destructor* [10], which is the mite vector for bee pathogens. Taken together, these losses appear to be closely associated with reduced immunocompetence resulting from viruses or *V. destructor* feeding [2, 10–13] and, therefore, mechanisms to enhance immune function are likely to reduce infection rates and increase colony viability. While most current research is focused on describing the factors causing mortality, few studies aim to identify novel physiological pathways capable of mitigating the stress-induced damages to restore bee health. For instance, current work in the field aims to restore bee health by increasing forage diversity, describing nutrition deficits, reducing insecticide exposure, and eliminating other stressors that are known to reduce colony health. However, the translation of these data sets from “bench-to-field” is difficult to implement in more immediate timescales and, as such, the knowledge gained in these areas have limited effectiveness in increasing sustainability of managed colonies. Furthermore, previous efforts to develop therapeutics to prevent pathogen infection of honey bees has focused on genetic technologies, such as RNA-interference (RNAi), which were successful in field trials, but have had challenges being commercialized on a large scale [14–16]. This is evidenced by the recent Bee Informed Partnership report that shows a consistent rate of annual colony loss ranging from 40%, 51%, and 40% in 2019, 2020, and 2021, respectively [17, 18]. Thus, identification of physiological targets that can be translated from the laboratory to the colony level in apiaries is needed to increase colony sustainability and was, therefore, the objective of this study.

One of the primary bee antiviral defense mechanisms is an evolutionarily conserved gene silencing mechanism known as RNA interference (RNAi), which recognizes the presence of double-stranded RNA to initiate targeted degradation of viruses [19–21]. Little information exists regarding mechanisms to enhance RNAi pathways in bees, but previous work has demonstrated that a subfamily of inward rectifier potassium (Kir) ion channels, termed ATP-sensitive inward rectifier potassium (K_ATP_) channels, play a significant role in mediating the survival of both mammals and flies during a viral infection through modulation of antiviral RNA interference in the heart tissue [22–24]. Importantly, our previous work has shown the functional coupling of K_ATP_ channels to antiviral immunity is conserved in bees, as we documented pharmacological activation of K_ATP_ channels significantly reduced bee mortality and virus mRNA replication after inoculation with Flock House virus (FHV), an entomopathogenic and cardiotropic virus that has not co-evolved with bees [25]. These data, coupled with our previous work indicating K_ATP_ channels regulate contractility of bee cardiac tissue [26], suggest heart specific regulation of antiviral RNAi pathways in bees can protect against infection with cardiotropic viruses. However, the applied relevance of these studies remained unknown due to the use of a model insect virus and the absence of field studies. Thus, it was important to determine the functional relationship between K_ATP_ channels and viruses that co-evolved with individual bees in the laboratory and at the colony level in the field, as well as define the mechanism of virus resistance.

While the physiological and toxicological relevance of Kir channels is becoming realized within arthropods of medical and veterinary relevance [24, 27–37], little is known about the role of the family of Kir channels in bees and this gap in knowledge has restricted our ability to define their fundamental roles in bee health. Interestingly, K_ATP_ channels are coupled to the metabolic state of the cell that enables these channels to regulate levels of reactive oxygen species (ROS) through uncoupling of the mitochondria [38–42], which provides a protective role against hypoxic stress in *Drosophila melanogaster* [43] and cockroaches [44]. Further, ROS are also critical to animal survival as they can function as signal messengers for the immune system after virus infection and, thus, a moderate increase in ROS has been shown to benefit animal health by enhancing immune function [22, 23, 43–46]. This suggests that a functional linkage between K_ATP_ channels, ROS, and antiviral defense mechanisms may also exist in bees. Therefore, we directly tested the hypotheses that K_ATP_ channels modulate ROS levels in individual bees and are amendable to pharmacological modulation to reduce bee mortality after inoculation with Israeli Acute Paralysis virus (IAPV), which is an impactful bee relevant virus, previously linked to ‘colony collapse disorder’ [2, 47]. Further, we show pharmacological activation of K_ATP_ channels nearly eliminate virus infection at the colony level in field apiaries to titers that were not significantly different than control colonies. These data are the first evidence of pharmacological therapies that reduce viral infections at the apiary level and validate K_ATP_ channels as a pharmacologically relevant target that can protect bees and restore healthy apiaries through inhibiting viral infection of mature colonies in the field.

## Results

### K_ATP_ modulators are non-toxic to adult bees

**Fig. 1:**
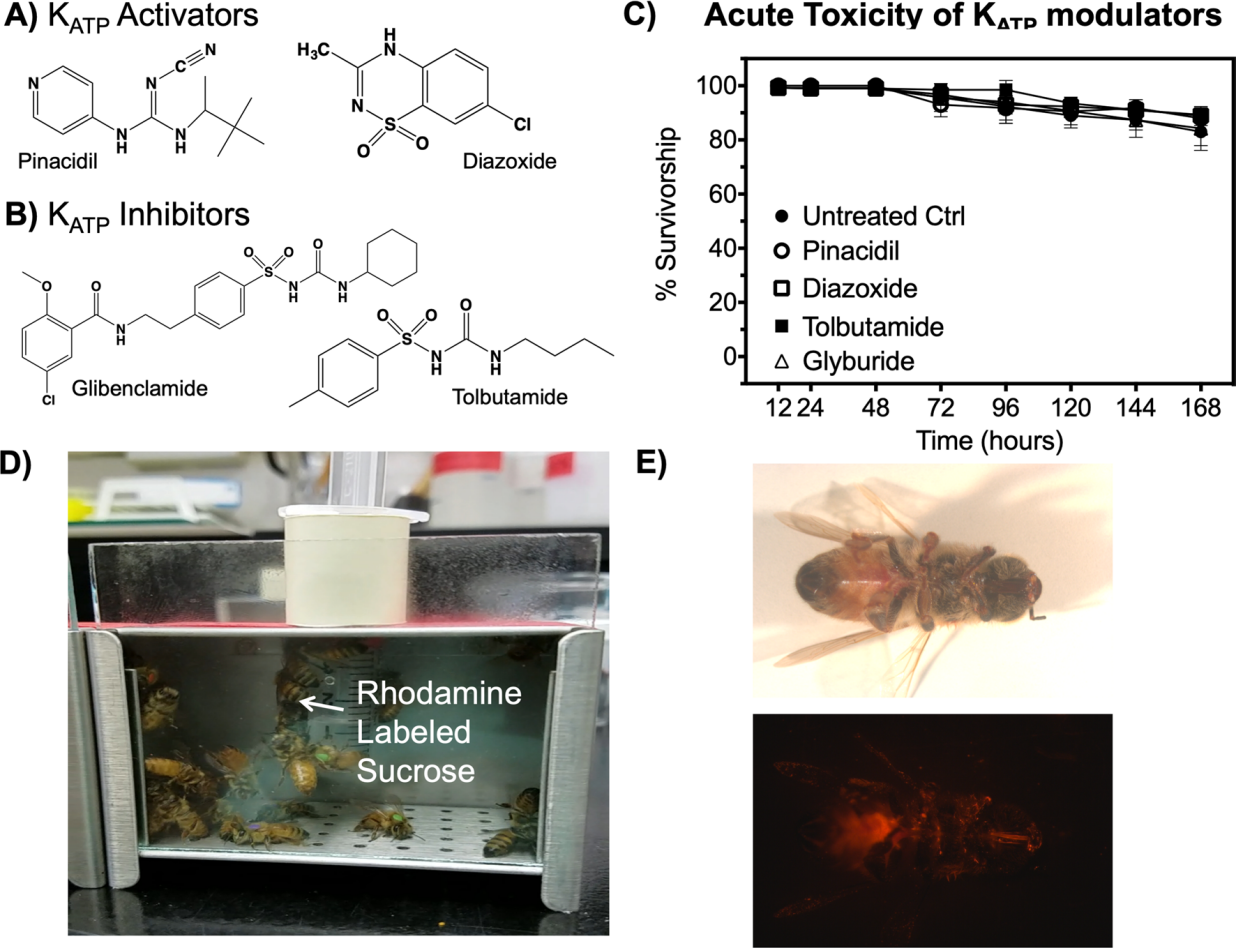
Structures of K_ATP_ modulators and their toxicity to bees. K_ATP_ channel activators (**A**) and inhibitors (**B**) used in this study. **C** Toxicity of K_ATP_ modulators to honey bees. Data points represent percent mean (n = 5, 50 bees per replicate survivorship with error bars representing SEM. **D** Image of caged bees with sucrose solution containing Rhodamine B and chemical modulators. **E** Representative image of bees under white and fluorescent light that verify individuals fed on the chemical treated solution

Prior to studying the physiological function of bee K_ATP_ channels using pharmacological activators (Fig. 1A) and inhibitors (Fig. 1B), we tested their oral toxicity to bees from the same colony by providing 50 caged individuals access to the compounds delivered in sugar water for 1 week. Compounds were tested at concentrations approaching solubility limits (2–4 mM) and data shown in Fig. 1C clearly indicate that chronic, oral exposure to these molecules is not acutely toxic. To ensure the bees ingested the chemical solutions, we treated the solution with the fluorophore, Rhodamine B, and only fluorescent bees were included in the analysis (Fig. 1D, E). The lack of toxicity suggests that using these modulators to probe the physiological function of K_ATP_ channels in bees is a viable approach.

### K_ATP_ channels regulate IAPV replication in bees

**Fig. 2:**
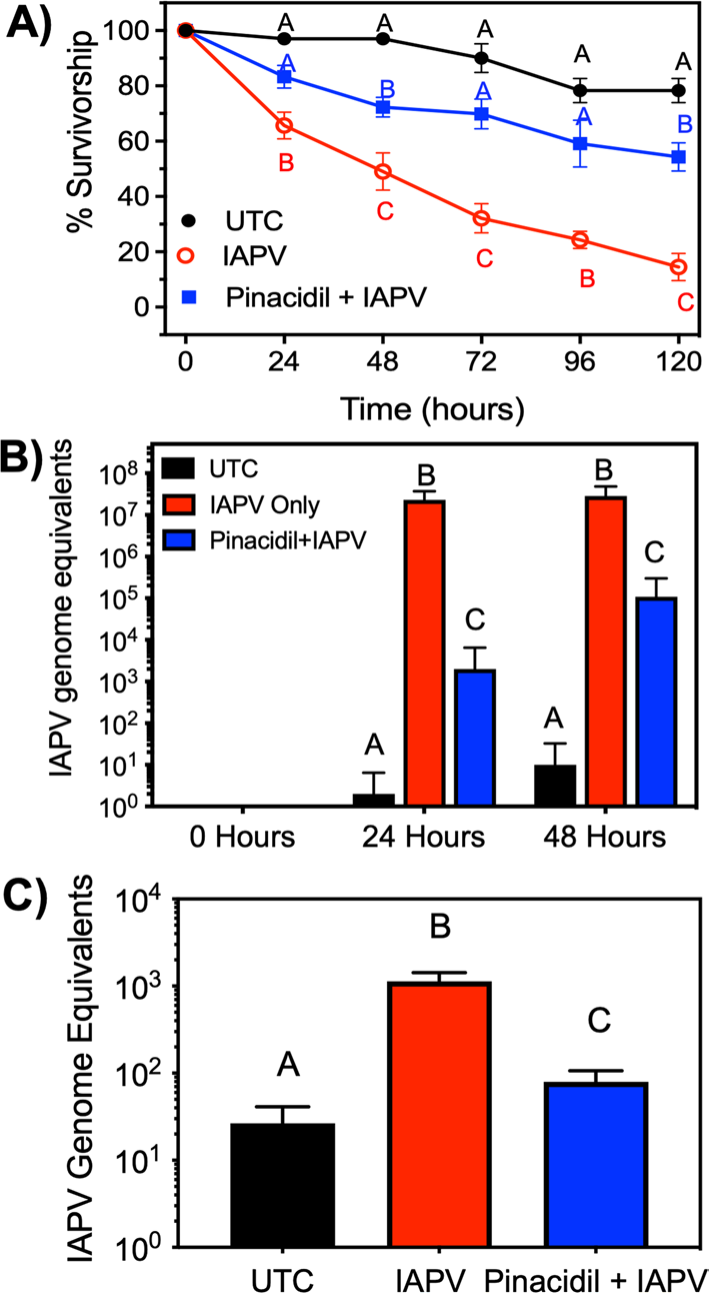
Pinacidil reduces IAPV-mediated mortality and IAPV replication in bees. **A** Data presented as Kaplan–Meier survival curves with data points representing mean survivorship with error bars representing SEM (*n* = 5, 30 individuals per replicate). Data points for each time point with different letters represent statistical significance as determined by Kaplan–Meier log-rank test with significance designated at *P* < 0.05. Bees were injected with 26 GEU of IAPV for IAPV only and pinacidil + IAPV groups. **B** Log_10_ fold change in IAPV mRNA expression over time following IAPV only or IAPV + pinacidil exposure from whole bees. Bars represent mean (n = 10, 5 individuals per replicate) IAPV genome equivalents. IAPV transcript expression from homogenized bees and error bars represent SEM. **C** Change in IAPV mRNA expression in dorsal vessels excised from bees 48 h post-infection. Bars represent mean transcript expression and error bars represent SEM (*n* = 25, 3 individuals per replicate). For **B**–**C**, Bars not labeled by the same letters represents statistical significance at *P* < 0.05 as determined by one-way ANOVA and Tukey’s post

We monitored IAPV mRNA in bees exposed to the K_ATP_ channel activator pinacidil and/or IAPV alone to test if potassium efflux through K_ATP_ channels regulates IAPV infection via virus replication or other steps of the viral cycle. We observed IAPV exposure to result in 35% and 85% mortality at 24- and 120 h post-inoculation, respectively, which are significantly (*P* < 0.05) higher than observed for the control bees (Fig. 2A, red trace). Bees provided pinacidil were observed to have 1.4- to 3.0-fold higher survivorship after IAPV inoculation for 24–120 h, which is significantly higher (*P* < 0.05) than bees surviving IAPV alone 24–120 h post-inoculation (Fig. 2A, blue trace). No significant difference in mortality was observed between pinacidil + IAPV and control treatments at 24-, 72-, or 96-h post-inoculation. However, bees exposed to pinacidil + IAPV inoculation for 48 and 120 h were observed to have a significant 25% reduction (*P* < 0.05) in mortality compared to control bees marking the largest difference between these groups (Fig. 2A). The ability to rescue survivorship via pinacidil exposure led us to hypothesize that K_ATP_ channels are involved in resistance to IAPV infection via reduced viral replication. Thus, we microinjected newly emerged bees with 26 genome equivalent units (GEU), or viral particles, of IAPV and quantified IAPV mRNA titers daily in the absence and presence of pinacidil. Pinacidil was found to reduce IAPV replication, based on the finding that IAPV mRNA was significantly (*P* < 0.05) reduced by 4.3-log units (11,471-fold) at 24 h post-infection and 3.5 log units (261-fold) at 48 h post-infection (Fig. 2B). These data are noteworthy as they provide evidence that the K_ATP_ activator pinacidil can protect bees from IAPV infection and prevent IAPV-induced mortality.

### K_ATP_ modulation reduces cardiotropic IAPV mRNA in bees

Tissue tropisms of IAPV are not fully defined and, thus, we aimed to test the hypothesis that IAPV is a cardiotropic virus. We detected IAPV mRNA at a titer of 1.1e3 ± 0.3e3 genome equivalents in individual dorsal vessels excised 24 h post-inoculation, a significant (*P* < 0.05) 43-fold increase in virus titer compared to bees not inoculated (Fig. 2C). Importantly, bees exposed to pinacidil 24 h prior to IAPV inoculation showed a significant (*P* < 0.05) 17.5-fold reduction in IAPV mRNA titer in the dorsal vessel compared to IAPV-inoculated bees not exposed to pinacidil (Fig. 2C).

### K_ATP_ channel activators increase ROS production in bees

**Fig. 3:**
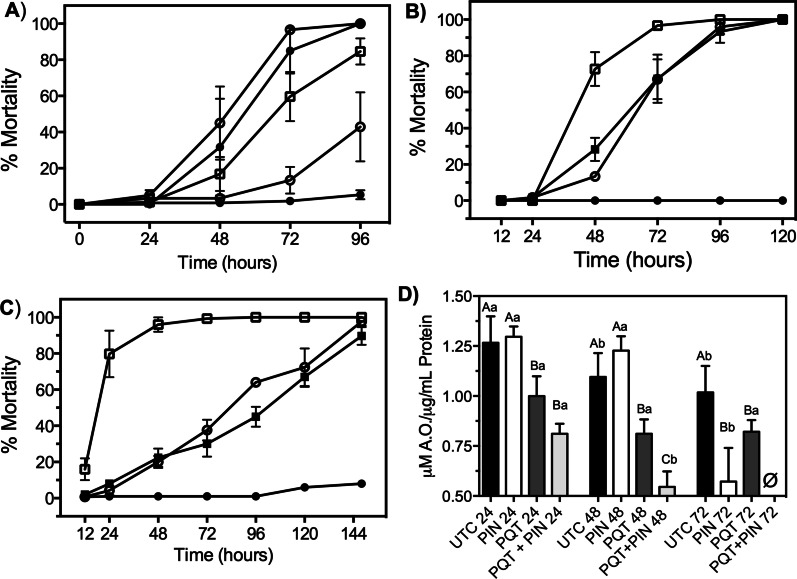
Pinacidil and tolbutamide modulate paraquat-induced mortality of bees. **A** Concentration–response of paraquat-mediated mortality to honey bees over time. Data points represent mean (n = 5, with 25 individuals per replicate) mortality and error bars represent SEM. Changes to mortality with pinacidil was assessed with routes of exposure being oral- (**B**) and injection (**C**). Mortality was assessed at 24-h intervals and data points represent mean (n = 75 bees) % mortality. Error bars represent SD. **D** Change in total antioxidant capacity over the course of 72 h after exposure to paraquat alone or paraquat/pinacidil combination. Bars represent mean (n = 50–100 bees) antioxidant levels and error bars represent SD. Uppercase letters represent statistical significance (*P* < 0.05) within the same time point and lowercase levels represent statistical significance (*P* < 0.05) of the same chemical treatment group across the time points. Significance was determined by one-way ANOVA with Tukey’s posttest

To define the functional mechanism of reduced IAPV-mediated mortality after exposure to K_ATP_ modulators, we examined the role of K_ATP_ channel regulation of ROS in bees. It is standard protocol to induce ROS in bees via injection of paraquat, an herbicide that elicits reproducible levels of ROS whereas other measures of ROS production are highly variable in bees [48]. Thus, we measured bee mortality after exposure to paraquat at concentrations of 0.1–2 μg/mL (Fig. 3A). Paraquat at 0.5 μg/mL showed slower onset of toxicity to bees, where we observed *ca.* 20% and 58% mortality at 48- and 72-h post-exposure, respectively (Fig. 3A). Paraquat at 1 and 2 mg/mL resulted in *ca.* 50% mortality at 48 h post-exposure and *ca.* 100% mortality at 72 h post-exposure (Fig. 3A). Therefore, we chose paraquat at 0.5 μg/mL to be suitable for testing the functional linkage between K_ATP_ channels and ROS due to the slower onset of toxicity compared to higher concentrations of paraquat.

We aimed to determine the influence of K_ATP_ channel activators and inhibitors on bee mortality resulting from paraquat-induced ROS. We observed that pre-exposure to the K_ATP_ channel inhibitor tolbutamide did not alter paraquat-induced toxicity over a 120-h feeding period (Fig. 3B, C). The K_ATP_ activator pinacidil with co-exposure to paraquat at 0.5 mg/mL significantly increased the rate of mortality resulting in an LT_50_ of *ca*. 40 h (Fig. 3B). However, paraquat exposure alone resulted in a significant (*P* < 0.05) increase in mortality, with a LT_50_ of *ca*. 68 h (Fig. 3B). To ensure the increased rate of mortality after pinacidil exposure was not an artifact of ingestion, we microinjected K_ATP_ modulators into bees that were orally treated with paraquat. The pattern of mortality remained the same with pinacidil increasing the rate of mortality, whereas K_ATP_ inhibitors did not alter paraquat-induced mortality. The rate of mortality was increased when compared to oral exposure with the pinacidil + paraquat combination resulting in an LT_50_ of *ca.* 20 h whereas 0.5 mg/mL paraquat alone resulted in an LT_50_ of *ca.* 75 h (Fig. 3C).

To functionally link pinacidil to ROS regulation in bees, we measured changes in total antioxidant concentrations in bees that were orally exposed to paraquat 24 h post-exposure to pinacidil. Pinacidil alone did not reduce antioxidant levels in bees when compared to untreated individuals at 24 h post-treatment (Fig. 3D). However, paraquat alone and paraquat in combination with pinacidil significantly (*P* < 0.05) reduced total antioxidant concentrations in bees by 1.3- and 1.9-fold, respectively, compared to untreated individuals. After 48 h, total antioxidant concentrations were not significantly different than the control treatment, yet paraquat significantly (*P* < 0.05) reduced total antioxidant concentrations by 1.4-fold when compared to the control treatment (Fig. 3D). Importantly, a binary combination of pinacidil and paraquat resulted in a significant (*P* < 0.05) reduction in total antioxidant concentration in bees by 3.0- and 1.7-fold after 48 h compared to untreated or paraquat-treated individuals, respectively (Fig. 3D). Conversely, pinacidil alone was found to significantly (*P* < 0.05) reduce total antioxidant concentrations by 1.5-fold after 72 h but was not different from paraquat alone after 72 h (Fig. 3D). There are no data for paraquat and pinacidil binary treatment of bees after 72 h due to greater than 95% mortality. Interestingly, the total antioxidant concentrations in bees after paraquat and pinacidil binary treatment were significantly (*P* < 0.05) reduced every 24 h, whereas bees exposed to paraquat alone did not have different total antioxidant concentrations after 24-, 48-, and 72-h (Fig. 3D). Collectively, these data indicate that pharmacological activation of K_ATP_ channels with pinacidil reduces antioxidants, which is usually due to increased ROS production [49], and suggests K_ATP_ channels can regulate ROS production in bees.

### ROS generation protects honey bees against IAPV infection

**Fig. 4:**
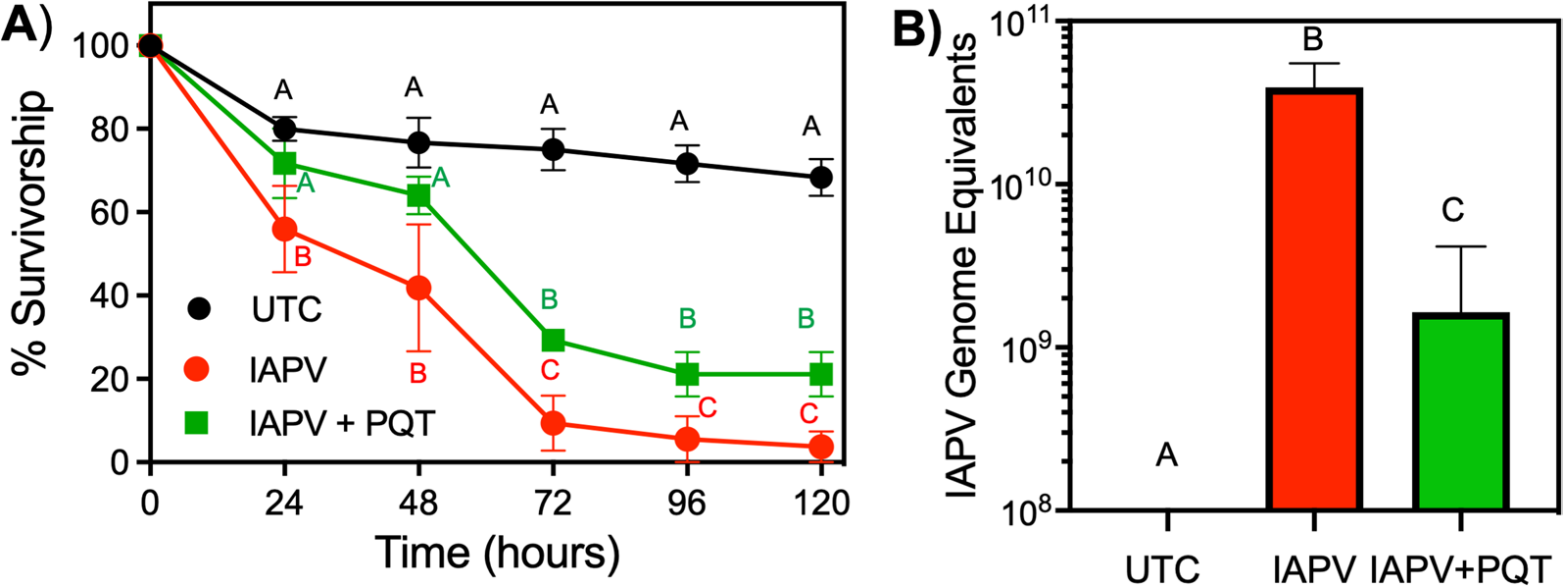
Paraquat reduces IAPV-mediated mortality and IAPV replication of bees. **A** Data presented as daily survival curves with data points representing mean (n = 5, 30 individuals per replicate) survivorship with error bars representing SEM. Data points for each time point that are not labeled by the same letter represent statistical significance as determined by Kaplan–Meier log-rank test) with significance designated at *P* < 0.05. Bees were injected with 26 PFU of IAPV for IAPV only and paraquat + IAPV groups. **B** Log_10_ fold change in IAPV mRNA expression over time following IAPV only or IAPV + paraquat exposure from whole bees. Bars represent mean (n = 10, 5 individuals per replicate)

To continue investigating the correlation between increased ROS and anti-IAPV infection, we tested if paraquat exposure will reduce IAPV infection in individual bees. IAPV exposure induced a significant (*P* < 0.05) 34% increase in mortality at 24 h post-inoculation, which significantly (*P* < 0.05) increased to 97% mortality at 120 h post-inoculation, when compared to the untreated bees (Fig. 2A, red trace). Bees that were provided 0.5 mg/mL paraquat, which was shown to induce significant levels of ROS (Fig. 3D), displayed increased survivorship after inoculation with IAPV when compared to IAPV alone. At 24- and 48-h post-inoculation, mortality in the IAPV + PQT binary treatment group was not significantly different than the untreated control mortality and was significantly reduced when compared to IAPV-alone (Fig. 4A). However, the protective influence of paraquat was lost at 72 h post-inoculation with paraquat + IAPV binary treatment mortality being significantly (*P* < 0.05) greater than untreated bees, but significantly less than IAPV-only treated bees (Fig. 4A). To correlate reduced IAPV-mediated mortality to reduced viral replication, we needle-inoculated newly emerged bees with 26 GEU IAPV and quantified IAPV mRNA titers in the absence or presence of paraquat. The IAPV mRNA titers of paraquat-treated bees were significantly reduced (*P* < 0.05) by 1.5-log units (24.3-fold) at 24 h post-inoculation (Fig. 4B).

### ROS reduces the rate of bee mortality in colonies heavily infested with *Varroa destructor*

**Fig. 5:**
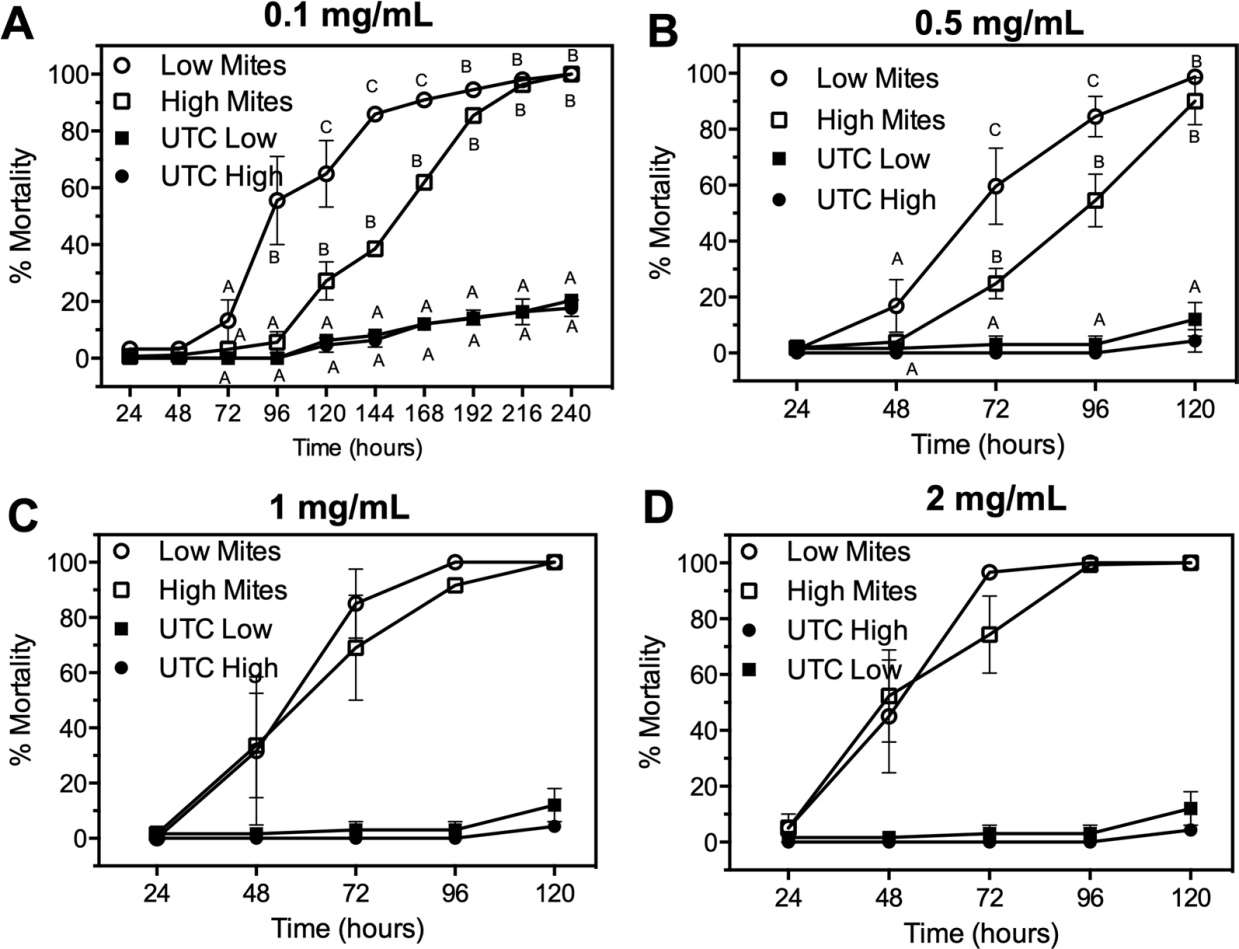
Paraquat mortality for bees from high- and low-mite infested colonies. Honey bees from low (circles) or high (squares) mite colonies were provided access to 0.1 mg/mL (**A**), 0.5 mg/mL (**B**), 1 mg/mL (**C**), or 2 mg/mL (**D**) paraquat and mortality at 24-h intervals. Data points represent percent mean (*n* = 75 bees) mortality and error bars represent SD. For panels **A**–**B**, data points in the same time point that are not labeled by the same letter represent statistical significance at *P* < 0.05 as determined by a one-way ANOVA with Tukey’s posttest

We aimed to test if ROS stimulates bee immunity in a field-relevant experiment with mite infestations of bees. We collected bees from high (> 10 mites/100 bees) and low (< 1 mite/100 bees) mite-infested colonies. The bees were orally exposed to paraquat concentrations reported to elicit injurious effects via ROS [50–52]. The premise is that bees from high mite-infested colonies have higher virus titers [53, 54] and ROS-mediated activation of immune responses might be beneficial to bees. Paraquat at a concentration of 0.1 mg/mL significantly increased (*P* < 0.05) the survivorship of bees from high mite-infested colonies compared to those from low mite-infested colonies at all time points from 96- to 168-h post treatment, but was lost at 192+ h post treatment (Fig. 5A). At a concentration of 0.5 mg/mL paraquat, significant (*P* < 0.05) reduction of mortality was observed in high mite colonies compared to low mite colonies at 72- and 96-h post treatment but was lost at 120 h post treatment (Fig. 5B). There were no statistical differences in survivorship between low and high mite treatment groups after exposure to 1 and 2 mg/mL paraquat (Fig. 5C, D).

To enable statistical comparison of high- and low-mite data, we determined the time required to achieve 50% mortality (LT_50_) for each concentration of paraquat. The largest difference in LT_50_ values was found with 0.1 mg/mL paraquat, where a 2.1-fold difference between high- and low-mite colonies was observed (Table 1), which was statistically (*P* < 0.05) significant. The difference in LT_50_ values was reduced, although still significant (*P* < 0.05), among bees from colonies with high mite infestations exposed to at 0.5 mg/mL paraquat with mortality 1.5-fold slower than bees from low-mite colonies. No significant difference in the rate of mortality between bees from high- and low-mite colonies was observed at paraquat concentrations of 1 mg/mL or higher (Table 1).

**Table 1:** Mortality of bees exposed to paraquat from low and high mite-infested colonies

	LT_50_ (Hours; 95% CI)	
	Low mites	High mites
0.1 mg/mL	82 (51–99) Aa	169 (142–177) Ba
0.5 mg/mL	66 (52–80) Ab	99 (71–136) Bb
1 mg/mL	54 (38–70) Ab	52 (22–84) Ac
2 mg/mL	49 (42–56) Ab	47 (38–63) Ac

Low mite-infested colonies were defined as < 3 mites/100 bees and high mite-infested colonies were defined as > 8 mites/100 bees. The uppercase letters represent statistical significance within the row (low vs. high mite) and lowercase letters represent statistical significance within the column (paraquat concentrations). Rows or columns not labeled by the same letter represents statistical significance at *P* < 0.05 as determined by a one-way analysis of variance (ANOVA) followed by Tukey’s post-test

LT_50_: time required to elicit 50% mortality

### Pinacidil stimulates bee social immunity markers, but not individual immunity markers

**Fig. 6:**
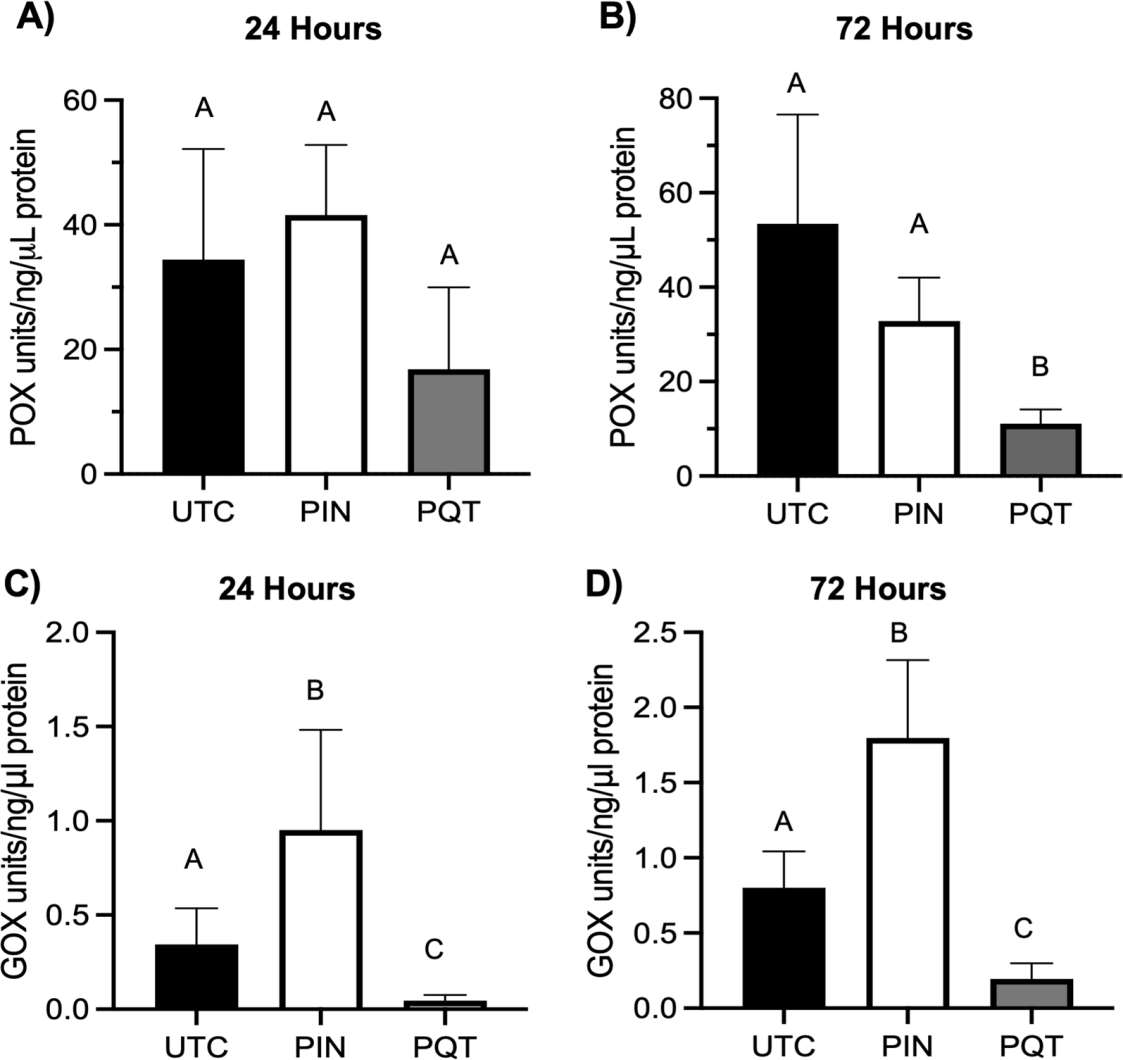
Phenoloxidase (POX) and glucose oxidase (GOX) activity of bees treated with pinacidil and paraquat. Pinacidil- (PIN) and paraquat- (PQT) treated nurse bees were examined for POX activity after 24 h (**A**) and 72 h (**B**) of treatment. PIN and PQT treated nurse bees were examined for GOX after 24 h (**C**) and 72 h (**D**) of treatment. Untreated controls (UTC) were provided access to pollen but were not exposed to chemicals. Bars represent mean POX or GOX activity and error bars represent SD (*n* = 5, with 25 individuals per replicate). Bars in the same panel different letters represent statistical significance at *P* < 0.05 as determined by a one-way ANOVA with Tukey’s posttest

We aimed to test the effects of pinacidil and paraquat on individual and social markers of immunity to determine the influence of potassium (K^+^) flux and ROS on bee immune health. The changes in phenoloxidase (POX) activity were used as a measure of individual immunity whereas changes in glucose oxidase (GOX) activity were used as a measure of social, or colony-level, immunity. Both represent constitutive mechanisms of immunity, and as such are metrics to assess general individual or colony-level investment in immune function based on specific conditions [55]. While there were no statistical differences in POX activity in bees after 24- and 72-h exposure to pinacidil, POX activity was significantly reduced (*P* < 0.05) in bees 72 h post-exposure to paraquat (Fig. 6A, B). Pinacidil significantly (*P* < 0.05) increased GOX activity in bees by 3- and 2.3-fold after 24- and 72-h exposure compared to untreated bees. However, paraquat significantly decreased (*P* < 0.05) GOX in bees by 11- and 3.3-fold compared to untreated and pinacidil-treated bees (Fig. 6C, D).

### “Bench-to-field” translation verifies pinacidil exposure eliminates virus replication at the colony level in the field

Translation of laboratory studies to increased health of bee colonies in the field has been a significant challenge and represents the limitation of most bee-focused research. Thus, we tested changes to replication of six viruses that have been attributed to declines in bee health through a field study that compared untreated, virus inoculated, and pinacidil + virus inoculated full sized, industry relevant colonies.

#### Validation of pinacidil exposure methods at the colony level

It was not known if bees would consume the same concentration of pinacidil-laced sucrose in the field as they do in the laboratory. We included the fluorophore rhodamine B at 1000 ppm to both untreated and pinacidil-laced syrup and compared consumption by quantifying\fluorescence of worker bees 24 h after exposure. Bees fed sucrose plus rhodamine B (untreated control) showed a significant (*P* < 0.05) 7.5-fold increase in fluorescence compared to sucrose alone (negative control), while bees fed sucrose containing pinacidil with rhodamine displayed a significant 40.3-fold increase in fluorescence compared to negative controls (Additional file 1: Fig. S2A). Pinacidil-exposed bees further displayed a significant 5.4-fold increase in fluorescence compared to untreated controls (Additional file 1: Fig. S2A). We additionally assessed freshly stored nectar near regions of comb containing young larvae. Nectar from colonies without- and with- pinacidil treatment displayed a significant 30.9- and 34.3-fold increase in fluorescence compared to colonies without rhodamine, which were not significantly different from each other but significantly (*P* < 0.05) increased from non-rhodamine treated colonies (Additional file 1: Fig. S2B). A representative abdomen of a bee after rhodamine B consumption is shown in Additional file 1: Fig. S2C.

#### DWV-A and DWV-B

**Fig. 7:**
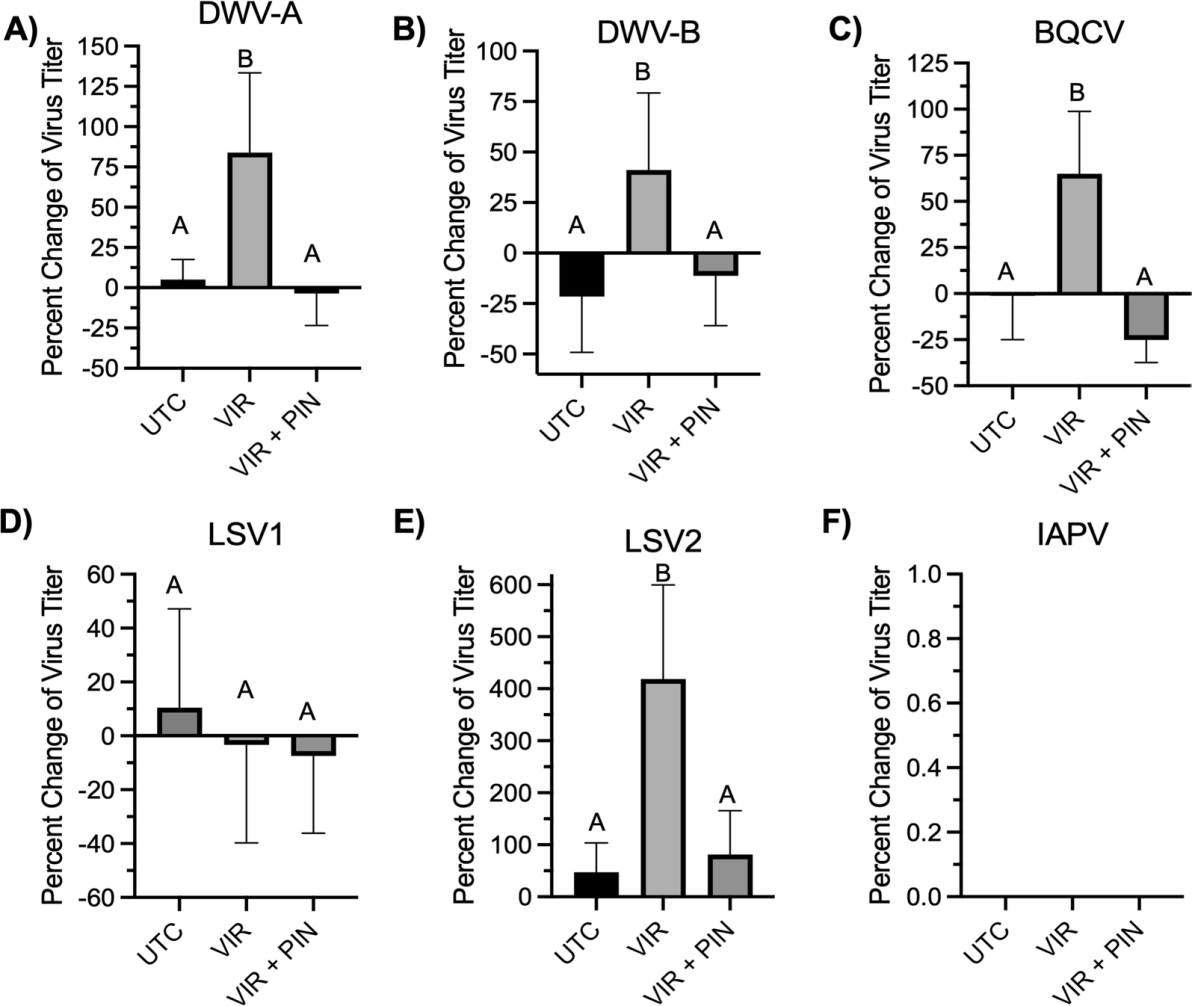
The effect of pinacidil exposure on virus infection in emerging bees in inoculated colonies. Viruses screened include DWV-A (**A**), DWV-B (**B**), BQCV (**C**), LSV1 (**D**), LSV2 (**E**), and IAPV (**F**). Bars represent average (n = 10 colonies) percent change in virus titer among emerging bees between initial and post-treatment timepoints and error bars represent SEM. Bars not labeled by the same letter within each virus group represents statistical significance at *P* < 0.05 as determined by a one-way ANOVA followed by Tukey’s posttest. Treatment groups include untreated (UTC), virus only (VIR), and 2 mM pinacidil + virus (VIR + PIN)

Changes to DWV-A or DWV-B titers in emerging brood, which were collected from individual cells that bees were actively emerging from, across the study period were significantly (*P* < 0.05) increased in inoculated colonies with statistical increases of 79% and 61% when compared to untreated colonies, respectively (Fig. 7A, B). Importantly, change of DWV-A or DWV-B titers in colonies exposed to pinacidil were eight- and sixfold reduced when compared to change in titers from virus only inoculated colonies and were not significantly different from uninoculated colonies (Fig. 7A, B). Interestingly, DWV-A and DWV-B titers in nurse/forager bees were not significantly different between virus only and pinacidil + virus treatments (Additional file 1: Fig. S3A–B).

#### Black queen cell virus (BQCV)

BQCV titers in colonies inoculated with BQCV was increased by 64% over uninoculated colonies, which was a statistically significant (*P* < 0.05) increase (Fig. 7C). Emerging brood in colonies treated with pinacidil had a significantly (*P* < 0.05) lower BQCV replication across the study period with treated colonies being 3.8-fold reduced when compared to virus only. The change in virus titer across the study period for pinacidil treated colonies was not significantly different than untreated colonies (Fig. 7C). However, pinacidil treatment did not reduce viral replication across the study period in nurse/forager bees compared to virus only inoculated colonies (Additional file 1: Fig. S3C).

#### Lake Sinai virus (LSV) 1 and 2

Colonies inoculated with virus did not show increase in LSV-1 across the study period for emerging brood (Fig. 7D) and only small amounts of LSV-1 replication was observed for nurse bees (Additional file 1: Fig. S3D). However, emerging brood in virus-inoculated colonies showed high levels of LSV-2 replication across the study period with a 418% increase compared to control colonies (Fig. 7D), yet little replication was observed in nurse bees (Additional file 1: Fig. S3D). As observed with other viruses, LSV-2 replication in emerging brood from colonies treated with pinacidil was reduced by approximately fivefold when compared to virus inoculation-only colonies, which was a significantly (*P* < 0.05) significant reduction (Fig. 7E). The lack of replication for LSV1 in emerging or nurse/forager bees and LSV-2 in nurse bees prevents statistical comparison among groups for these viruses.

#### IAPV

Comparisons across treatment groups are not possible for emerging brood as IAPV mRNA was below detectable limits in all groups (Fig. 7F). For nurse bees, IAPV was detected in control, but the low levels prevented comparison among groups (Additional file 1: Fig. S3F).

## Discussion

The consistent and unsustainable decline of managed honey bee colonies continues to plague the apiculture and agriculture industries despite concerted research efforts aiming to define the mechanisms driving the high rate of annual colony losses [17]. These declines are attributed to a myriad of factors, but virus infections are consistently considered to be a leading factor for excessive mortality of bee colonies. It is surprising that ‘druggable’ targets to enhance the antiviral immune response of bees have yet to be identified. Considering this, we aimed to test the toxicological relevance of K_ATP_ channels to reduce virus replication and virus-mediated mortality at the level of the individual bee and at the colony level. Data indicate activation of K_ATP_ channels reduce IAPV-replication and mortality to near control level in the laboratory. This result was successfully translated to the apiary with near elimination of infection with 6 viruses in full-sized colonies. Together, these data implicate K_ATP_ channels as a novel, pharmacologically relevant target for the development and deployment of antiviral therapies in apiaries to reduce virus infection and restore health across apiaries.

While it has been shown to be possible to reduce pathogen infection through development of molecular genetic technologies targeting the pathogen [14, 15], such as RNAi, the identification of therapeutically relevant targets in the honey bee to prevent infections at the apiary-level have remained unclear. Previous studies have defined molecular mechanisms of bees to reduce pathogens [20, 21, 56, 57] and have aimed to validate targets using molecular genetics that functionally link stress proteins to the antiviral defense of bees against a model Sindbis virus with the goal of reducing virus mortality [58]. These analyses have provided insights regarding genetic regulation of these systems, but actual production of immune factors has been understudied and thus, knowledge gaps remain regarding the complex relationship between bees and virus as well mechanisms to modulate this interaction with therapeutics. These knowledge gaps have limited our understanding of how viruses and immune deficiencies affect bee health and, in turn, restricts our ability to develop effective strategies to mitigate colony losses. Bees are known to utilize an antiviral RNAi response [59, 60], and other immune pathways, for protection from virus infections, but little information exists regarding the mechanisms bees use to initiate the RNAi response. Previous work has indicated K_ATP_ channels are involved in the antiviral response of flies as KIR2 knockdown and the pharmacological inhibition of K_ATP_ channel function leads to a strong increase of model virus replication resulting in accelerated death of virus-infected mammals [22], flies [22, 23] and bees [25]. These studies reveal an evolutionarily conserved relationship between K_ATP_ channels and the antiviral immune response via regulation of virus resistance mechanisms in the animal heart [22, 23, 25, 26]. However, questions remain regarding the involvement of K_ATP_ channels in antiviral resistance to non-model virus infection and, more specifically, the toxicological relevance of this pathway to mitigation of mortality resulting from viruses that have co-evolved with bees, such as IAPV. Thus, the goal of this work was to define the underpinnings of this linkage by determining the relationship between K_ATP_ channels, ROS generation, and survivorship in bees after challenge with IAPV, a virus that has co-evolved with bees and is of significant relevance to the apiculture industry [47, 61]. We then aimed to expand these fundamental data into industry-relevant data by testing the utility of this system in the field at the colony level.

A primary determinant of animal survival during virus infection is the innate immune response, which is responsible for mounting an interference response that prevents virus replication to regulate infection and disease. Potassium (K^+^) ion flux regulates antiviral immunity in mammals through inflammasomes, antiviral apoptosis, and RNAi pathways [22, 23, 62, 63]. The mechanism by which K^+^ ions regulate these antiviral pathways remains unclear, but it is known that: (1) K^+^ channels repolarize cardiac myocytes [64]; (2) K_ATP_ channels regulate infection of cardiotropic viruses [23]; and (3) mutation of genes encoding K^+^ efflux pathways led to higher virus titers in mammalian hearts [22]. Taken together, this suggests K^+^ ion efflux may regulate heart-specific antiviral defense mechanisms in mammals and insects. We previously reported pinacidil, an activator of K_ATP_ channels, exerted a protective effect against FHV infection of bees [25], similar to findings described for *Drosophila*, where K_ATP_ channels regulated cardiac function [26]. Here, we have expanded these proof-of-concept data into an evolutionarily aligned system by verifying that activation of K_ATP_ channels with pinacidil protects individual bees from IAPV infection and mortality. Thus, we aimed to test if the functional relationship between K_ATP_ channels and antiviral activity is driven by circulatory homeostasis resulting from activity at the bee dorsal vessel. Tissue tropisms of IAPV has been studied, but migration to honey bee dorsal vessel tissue was not addressed [47]. IAPV mRNA was detected in excised dorsal vessels that did not have associated alary muscles or body wall tissue (Fig. 2C), which indicates pinacidil-mediated reduction of IAPV replication could be due to pinacidil influencing dorsal vessel activity in bees. Previous work indicates K_ATP_ activators to stimulate dorsal vessel contraction rates in bees [26], which is relevant to our study because the insect dorsal vessel is a homeostatic tissue and insect cardiac function is directly correlated to infection dynamics [65, 66]. In mosquitoes, the dorsal vessel controls the damage resulting from a pathogen infection [22] and, more specifically, hemolymph currents are essential for systemic antiviral RNAi responses in insects [65, 67, 68]. This suggests increased dorsal vessel function may stimulate anti-pathogen responses and reduce susceptibility to virus infection, but the applicability of this correlation to insects other than mosquitoes previously remained unknown. The data presented in this study, combined with our previous work [25, 26], supports this correlation in bees with the verification that pinacidil: (1) increases dorsal vessel contraction rates [26]; (2) reduces mortality; (3) reduces IAPV replication; and (4) reduces IAPV transcripts in the dorsal vessel of bees (Fig. 2A, C). Although the inhibition of IAPV replication is clear, the physiological mechanism facilitating this reduction of IAPV infection and mortality required elucidation. Thus, we aimed to next test the hypothesis that K_ATP_ channels regulate IAPV replication via indirect control of reactive oxygen species (ROS).

Viral infections can increase levels of oxidative stress and, importantly, modulation of ROS levels decreases pathogenesis and virus replication [69]. Thus, ROS are considered putative antiviral targets to reduce the health burden of virus infection in humans [70]. ROS are well-documented to cause a number of deleterious events that lead to mortality of bees [71, 72] resulting from increased cellular damage [73]. It is understandable that many ROS studies have focused on the deleterious consequences of ROS to bee health, but ROS are also critical for animal survival. ROS function as signal messengers for the immune system after virus infection and, thus, a moderate increase in ROS benefits human health by enhancing immune function [45]. Furthermore, ROS are lethal to mosquitoes at high concentrations, but a moderate increase in ROS leads to higher mosquito survival after infection with bacterial or viral pathogens by enhancing immune responses [74, 75]. Thus, the biological effects of ROS likely operate around a hermetic scale that provides opportunities to harness the beneficial properties of ROS to enhance bee health. Indeed, paraquat-induced ROS was found to operate on a hermetic scale with 0.1 mg/mL paraquat stimulating appropriate levels of ROS (Fig. 3) to result in reduced IAPV-mediated mortality and replication (Fig. 4). These data verified that ROS is a physiological messenger to the immune system of bees and mechanisms to stimulate secretion of ROS at the appropriate levels has potential to regulate antiviral immunity in bees. Prior to this study, there were knowledge gaps regarding physiological mechanisms that can be exploited to modulate ROS levels in bees, yet data presented in this study implicate K_ATP_ channels as putative targets to regulate ROS levels and reduce virus-mediated mortality and virus replication. K_ATP_ channels are coupled to the metabolic state of the cell that enables these channels to regulate levels of ROS in animals [38] and are used as a signaling mechanism for immune system enhancement after virus infection [22, 23, 43, 44, 46] in mammals. Exposure of bees to the K_ATP_ channel activator pinacidil enhanced survivorship after IAPV inoculation to a degree that was not significantly different from uninfected bees at most time points (Fig. 2). Together, these data provide strong support that a functional linkage between K_ATP_ channels, ROS, and antiviral defense mechanisms exists in bees and validate this system as intervention points to reduce viral infections.

To further link the anti-IAPV activity of pinacidil to ROS, we measured the influence of pinacidil and paraquat to individual- and colony-level immune responses by assessing changes to the immune response biomarkers glucose oxidase (GOX) and phenoloxidase (POX) [76]. Interestingly, pinacidil and paraquat had opposite actions to GOX and POX activity, with pinacidil increasing and paraquat decreasing GOX activity (Fig. 6B). GOX is a biomarker for social, or colony-level, immunity, and our results support the premise that pinacidil exposure allows for increased investment in the production of compounds that can enhance bee resilience to stressors while paraquat negatively influences this process. This in turn could be partly responsible for the reduction of IAPV-mediated bee mortality when bees were provided pinacidil (Fig. 2A). POX is a biomarker for individual immunity and responsible for elements of the cellular immune response, which is increased in bees faced with an immune challenge or insecticide exposure [76, 77]. However, the prophenoloxidase cascade is also considered a major aspect of constitutive immunity that impacts processes like activation of melanization [78]. Thus, the reduction of POX levels in paraquat treated bees and the lack of reduction of POX in the pinacidil treated bees is likely an indication that paraquat causes a physiological stress impacting the bees ability to invest in general immune function, while pinacidil has no such negative impact. Overall, the combined findings suggest that not only antiviral immunity, but generalized immune function is supported by K_ATP_ channel activation that is worth continued exploration.

The focus of work related to bee antiviral treatments has been focused on the development of RNAi-based technologies against the pathogen, which has had difficulty in being translated to commercialization of field relevant applications [14–16]. Thus, we aimed to determine the field relevance and practicality of pharmacological modulation of ion channels to reduce virus replication in mature colonies through a large-scale field study. Our data verified antiviral activity of pinacidil through near elimination of viral replication in emerging bees, which provide strong support that it is indeed possible to reduce virus replication at the colony level after treatment with pharmacological activator of K_ATP_ channels. Importantly, the data also indicate that the antiviral capacity of this system is broadly applicable to viruses and is not virus species specific as we observed a near elimination of DWV-A, DWV-B, LSV-1, LSV-2, and BQCV in field studies and IAPV in laboratory studies (Figs. 2 and 7). While these data provide strong proof-of-concept, it is important to note that pinacidil did not reduce viral replication in nurse bees (Additional file 1: Fig. S3), which suggests exposure period is critical for activity of pinacidil and ROS to inhibit virus replication. Given the time period of sampling and treatment, it is likely that the nurse aged bees sampled for assessment may not have been exposed to pinacidil themselves. Nurse-aged bees would typically be aged 7–14 days at the time of collection and would likely have emerged around day 10 on the treatment schedule (Additional file 1: Fig. S1). As such, some may have been to exposed to the treatment early in their development but most of them likely would not have been exposed to chemical treatment, yet these bees would have received viral inoculum from what was on the comb or been infected by other workers. It appears that the strength of the viral inoculum may have overwhelmed the therapeutic benefit of the K_ATP_ channel activation by pinacidil in the exposed adults, but that the pre-treatment with pinacidil improved larval resistance to viral infection. Preventing infection in larvae is critical to reduce development of symptoms of viruses like DWV that cause lethal malformations during pupation but only result in sublethal effects when infected as adults. Future work should continue to explore the timing and longevity of treatment under conditions of natural viral infection.

In conclusion, the data presented here have revealed an important role for K^+^ ion channel regulation of the bee antiviral immune response by providing a putative linkage between K_ATP_ channels, ROS, and antiviral immunity. The ability to reduce IAPV replication and IAPV-mediated mortality via pharmacological modulation of ion channels versus direct action to the virus or bee immune system to inactivate bee viruses represents an original avenue to reduce virus-mediated losses of managed bee colonies. Importantly, our field data strongly support the notion that pharmacology can be used to reduce virus mediated mortality in full sized colonies, which expands previous demonstrations that RNAi strategies targeting the pathogen can curb the negative effect of IAPV infection at the colony level. Together, these data provide critical proof-of-concept for the development of therapeutics targeting ion channels that can inhibit virus-mediated mortality of bees to ultimately increase the sustainability of managed bees. Downstream efforts to develop novel therapeutics directed to regulatory mechanisms of the bee immune system should be considered.

## Materials and methods

### Pharmacological modulators and reagents

Paraquat dichloride hydrate (PESTANAL, analytical grade), K_ATP_ channel activators and inhibitors, rhodamine B, L-DOPA, d-glucose, *o*-dianisidine dihydrochloride, horse radish peroxidase and dimethyl sulfoxide (DMSO) were obtained from Sigma-Aldrich. All compounds were designated to be > 98% pure. Phosphate-buffered saline (PBS, 1×, pH 7.4) was obtained from ThermoFisher Scientific. White granulated sugar was purchased from Walmart Stores.

### Honey bee colonies

Honey bee colonies of commercial Italian stock were sourced from the Swale Lab apiary at Louisiana State University in Baton Rouge Louisiana. All in vitro studies were performed between the months of April-June and frames of capped brood near emergence were cleared of adult bees and transferred to an incubator (32 °C, ~ 70% RH, total darkness). Age-matched bees were collected 24 h later. All field studies were performed between the months of March–May to ensure low mite populations. For field studies, nucleus colonies of the same stock were established in eight frame Langstroth equipment in an isolated apiary. All nucleus colonies possessed five frames of bees, one frame of eggs and larvae, two frames of capped brood, one frame of honey and pollen, and one frame of drawn empty comb. After remaining queenless for 24 h, each nucleus colony was provided with a mated sister queen (Ramirez Brothers Apiaries, Modesto, CA, USA). Nucleus colonies used in field trials were randomly assigned to each for four treatment groups, including untreated control (sucrose diet, untreated inoculum), virus only (sucrose diet + viral inoculum), and virus plus pinacidil (2 mM pinacidil diet, viral inoculum). Finally, to minimize the transfer of pinacidil treatment solution or virus inoculum between treatment groups, colonies were outfitted with robbing screens (Mann Lake Bee and Ag Supply). For all trials, colonies possessed less than 1 *Varroa* mite per 100 bees as determined by alcohol wash, and no mite treatments were made during the experimental phase of any given trial. It is important to note that no chemical miticide treatment was used in the colonies and the low mite numbers were likely due to the early time of year (March–April).

### Evaluation of acute toxicity of KATP modulators to adult honey bees

Groups of 50 age-matched bees were transferred to glass and steel hoarding cages [79]. Stock solutions of pinacidil, diazoxide, glibenclamide and tolbutamide were made near solubility limits (200–400 mM) in 99.9% DMSO, then diluted 100-fold in 1:1 sucrose. Following methods modified from the OECD 213 guideline, bees were exposed to K_ATP_ modulators via ad libitum feeding for 168 h with mortality assessments at 24-h intervals [80]. Toxicity assessments were replicated five times with 50 age-matched bees per replicate.

### IAPV preparation

IAPV was obtained following our previously described methods [81]. Approximately 30 adult bees from a heavily infected IAPV infected colony were froze in liquid nitrogen, ground to a fine powder, and homogenized in a phosphate extraction buffer. The supernatant was collected after centrifuging the emulsion and filtered through a 0.2 micron filter. The filtrate was subsequently centrifuged at 30,000 rpm to pellet the picornavirus particles and was followed by resusopension in PBS and centrifuged at 52,000 rpm overnight. The fractions containing virus particles were dialyzed against cold PBS overnight. Verification that IAPV was the only virus present in the inoculum was performed via qRT-PCR and quantification of IAPV was performed by absolute quantification using standard curve method as previously described [82].

### Testing the role of KATP modulation and ROS induction to IAPV mediated mortality and replication

Groups of 30 age-matched bees were fed 1:1 sucrose diet alone or containing K_ATP_ modulators or 0.5 mg/mL paraquat for 24 h. Bees were immobilized on ice, then injected with PBS alone or containing IAPV and returned to the incubator. Mortality assessments and sampling of survivors were conducted at 24-h intervals. Quantification of IAPV mRNA among whole bees and excised insect dorsal vessels was performed by qRT-PCR as detailed below.

### Quantification of ROS

An LC_50_ for paraquat in 1:1 sucrose diet was determined for 72 h of oral exposure by constructing a concentration–response curve. The ability of K_ATP_ modulators to rescue or synergize paraquat-induced mortality was then assessed by incorporating modulators into 1:1 sucrose containing paraquat. Mortality assessments and sampling of surviving bees was conducted at 24-h intervals. Quantification of ROS among survivors was performed using the Total Antioxidant Capacity Assay kit (Cayman Chemical).

### Validating the therapeutic benefit of ROS modulation under field-relevant conditions

Age-matched bees from colonies with higher (> 10 mites/100 bees) and lower (< 1 mite/100 bees) were placed in hoarding cages and allowed to feed ad libitum on sucrose solution alone or containing paraquat. Survivorship was assessed at 24-h intervals, and LT_50_ times were calculated by nonlinear regression using a Hill equation.

### Biochemical assessments of GOX and POX activity

Groups of 50 age-matched bees were collected and exposed to sucrose alone or containing pinacidil or paraquat. Individual bees were sampled at 24-h intervals and frozen in liquid nitrogen. Social immunity was measured by quantifying the metabolism of glucose to hydrogen peroxide by glucose oxidase [83]. Briefly, heads were homogenized in ice-cold extraction buffer then centrifuged at 15,000×*g*. Aliquots of supernatant were incubated with horse radish peroxidase and glucose at 37 °C, then combined with the chromogen *o-*dianisidine dihydrochloride. Absorbance was read at 430 nm over a period of 90 min and compared to sample blanks, lacking chromogen. To measure individual immunity, phenoloxidase activity was measured as described [83]. Abdomens were homogenized as and centrifuged as previously described. Supernatant was incubated at 37 °C, then combined with the chromogen L-Dopa. Absorbance was measured at 490 nm for 20 min, then compared to sample blanks lacking chromogen. To compare both glucose oxidase and phenol oxidase activity between samples, V_max_ was calculated using the Michaelis–Menten equation.

### Validation of drug exposure methods in the field

While honey bee consumption of pinacidil solution has previously been confirmed in vitro, it was necessary to ensure the consumption of pinacidil solution under field conditions. Nucleus colonies of identical stock and strength were established as previously described, then randomly assigned to each of the following treatment groups: negative control (1:1 sucrose only), positive control (1:1 sucrose + 1% DMSO and 1,000 ppm rhodamine B [29–31, 33, 84], and Pinacidil (1:1 sucrose + 1% DMSO and 1000 ppm rhodamine B with 2 mM pinacidil). To measure the relative amount of each solution consumed, 100 mL of each respective solution was drizzled between the frames of the colony directly on to bees to maximize consumption. Samples of worker bees were taken from all colonies 24 h after exposure. Additionally, fresh nectar was collected from cells adjacent to regions of comb containing larvae. Nectar and bee samples were stored at − 20 °C overnight, then imaged using the Leica Thunder microscope using a Rhodamine filter cube (excitation wavelength 540 nm, emission wavelength 625 nm) [29]. Fluorescence was quantified in the abdomens of whole bees to confirm feeding on pinacidil solution. Fresh nectar fluorescence intensity was quantified by drawing 1 μL aliquots of nectar into 10 μL glass capillaries (Drummond Scientific, City, State, Country). Fluorescence intensity was quantified using ImageJ.

### Testing the ability of KATP channel activation to reduce virus infection under field conditions

Nucleus colonies of bees were established as previously described and relocated to a geographically isolated apiary and were grown to full-size colonies and equalized based on population prior to initiation of the study. The bees were maintained in eight-frame Langstroth equipment and kept as single deeps with a medium super with undrawn foundation. Pinacidil solution was prepared at 200 mM in DMSO, then diluted 100-fold in 1:1 sucrose. DWV inoculum was prepared by injecting filter-sterilized homogenate from a DWV-positive symptomatic bee into white-eyed honey bee pupae. 72-h later, an aliquot of pupae was screened for preliminary confirmation of DWV infection and found to contain between 5E9 and 8E9 viral copies of DWV-A per individual. Remaining pupae were homogenized in ice-cold PBS, with aliquots retained for qRT-PCR screening of DWV, as well as additional viruses relevant to bee health. Homogenate solutions were diluted in 1:1 sucrose and placed in calibrated spray bottles. A treatment and sampling schedule was constructed based on the 21-day egg-to-adult life history of worker bees (Additional file 1: Fig. S1). Both pinacidil and virus solutions were prepared fresh daily. Working under the illumination from red headlamps, 100 mL of sucrose alone or containing pinacidil was drizzled directly onto bees between the frames of each colony at 24-h intervals from day one to day nine of the treatment calendar. During the daylight hours, each frame of bees and brood was sprayed daily with 150 mL of sucrose alone or containing DWV inoculum on calendar days four, five and six. Samples of emerging and nurse/forager bees were taken on calendar days 1 and 21 (Additional file 1: Fig. S1). Nurse/forager bees were sampled by removing two brood frames from each colony, checking for the presence of the queen, and shaking each frame into a 5-gallon bucket. A ½ cup sample was then taken, equaling approximately 300 bees. To sample emerging bees, frames of capped brood with visibly chewed cells were cleared of all adult bees an taken to a nearby screened enclosure. Using soft-touch forceps, the capping of each cell was removed. A quantity of 15 fully formed adult stage bees were removed from the capped cells and placed into 15 mL conical vials. All samples were stored on dry ice during field collection, then transferred to − 80 °C until virus quantification. Titers of DWV-A, DWV-B, BQCV, IAPV, LSV-1 and LSV-2 were quantified by qRT-PCR using primer sequences described in our previous publications [85, 86].

### qRT-PCR quantification of virus titers

Samples were removed from − 80 °C storage and placed in a nuclease-free chilled bead bath (~ 4 °C) to gently thaw. For samples consisting of whole bees, pools of 30 bees (nurses) or 12 bees (newly emerging) were transferred to 30 mL screw-top vials containing 300 mg of 2.3 mm zirconium/ceramic beads. To each tube, 200 μL of ice-cold homogenization buffer was added (Promega Maxwell® RSC SimplyRNA Tissue kit). Tissue was homogenized by bead milling using the Omni Beadrupter Elite at 5 m/s for two cycles of 20 s with a 10 s pause between cycles for heat dissipation. Total RNA was then extracted using the Maxwell® RSC SimplyRNA Tissue kit and Maxwell® RSC instrument per manufacturer instructions. RNA concentrations were then quantified for each sample using the NanoDrop 1-C® instrument. To create cDNA libraries for each sample, 500 ng of RNA was transferred to a clean 0.2 mL tube with a variable amount of nuclease free water so that the final volume in each tube was 12 μL. Library construction was performed using the Quantitect Reverse Transcription kit according to manufacturer instructions (Qiagen, N.V.). Template cDNA was then quantified by NanoDrop 1-C® and standardized to 500 ng/uL by addition of nuclease-free H_2_O.

To accurately quantify virus infection, it was necessary to first construct a standard curve for each target virus. This was accomplished using an engineered PUC-57 plasmid containing the amplicon sequence for each set of PCR primers (GeneWiz/Azenta Inc.) The plasmid was then linearized using the Fast Digest EcoRV restriction enzyme (Eco 32I®, Thermofisher Scientific) according to manufacturer’s protocols. Linearized plasmid cDNA libraries were constructed using the SuperScript™ Vilo™ cDNA Synthesis Kit (Thermofisher Scientific) following a modified protocol that omitted DNA digestion steps. Single-stranded DNA concentration was quantified for each cDNA library with the NanoDrop 1-C® instrument (Thermofisher Scientific) and the number of amplicon copies per μL of solution was determined using online calculators available at SciencePrimer.com. Eight serial dilutions were made from the starting concentration of each plasmid cDNA library using nuclease free water, thus forming a standard curve relevant to each primer set. PCR primers were used as described in our previous publications [85, 86] and purchased from IDT (Integrated DNA Technologies, Inc.). Mastermix for quantification of each target was made using variable amounts of forward and reverse primer, SsoAdvanced™ Universal SYBR® Green SuperMix, and nuclease free water. The CFX Connect™ Real-Time PCR system (Bio-Rad Laboratories, Inc.) was used to quantify all viral targets using a 40-cycle protocol with included melt-curve analysis. Samples were run in triplicate and using the average CT values and known number of amplicon copies for each of eight plasmid standards, we performed a linear regression to quantify the number of viral copies present in unknown samples.

### Statistical methods

Description of statistical tests used are described in each figure legend. To compare mortality among paraquat exposed bees from colonies with higher and lower levels of *Varroa* infestation, differences in LT_50_ values were compared by unpaired T-test. For all other data types, means (including percent mortality, virus titer, antioxidant capacity, and enzyme activity) were compared by one-way analysis of variance (ANOVA) with Tukey’s posttest comparison. Significant difference defined as *α* = 0.05. All analyses were carried out in GraphPad Prism (version 9.0).

## Supplementary Information

**Additional file 1:** Addional figures supporting primary dataset.

## Data Availability

The datasets supporting the conclusions of this article (are) included within the article and supplementary materials.
