# Supplementary material for: Potassium ion channels as a molecular target to reduce virus infection and mortality of honey bee colonies
Source: Virol J. 2023 Jun 22;20:134. doi: 10.1186/s12985-023-02104-0 (PMC10286336; doi:10.1186/s12985-023-02104-0)
Supplement: Supplementary file 1 — Additional file 1: Addional figures supporting primary dataset. [file 12985_2023_2104_MOESM1_ESM.docx]

**Additional file**

**Title:** Potassium ion channels as a molecular target to reduce virus infection and mortality of honey bee colonies

**Authors:** Christopher J. Fellows^1^, Michael Simone-Finstrom^2^, Troy D. Anderson^3^, Daniel R. Swale^1,4 *^

**Additional file 1: Figures**

**
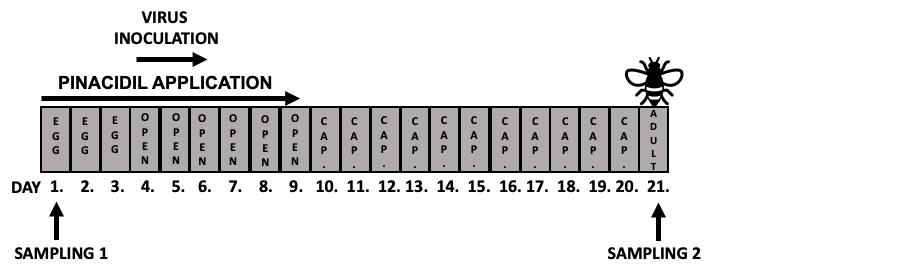
**

**Additional file 1: Fig. S1. Schedule of drug and virus applications to honey bee colonies.** Horizontal bars represent a hypothetical cell of comb containing a fertilized egg laid on day one. Text inside bars represents worker life stages including “egg”, “open” (larvae), “cap.” (capped stage, pre-pupa, and pupa), and “adult”. Pinacidil applications coincided with the “open” stages of development, while virus inoculation was timed to occur during the first three days of the larval stage. Sampling of nurse bees and emerging adults occurred on day 1 and day 21.

**
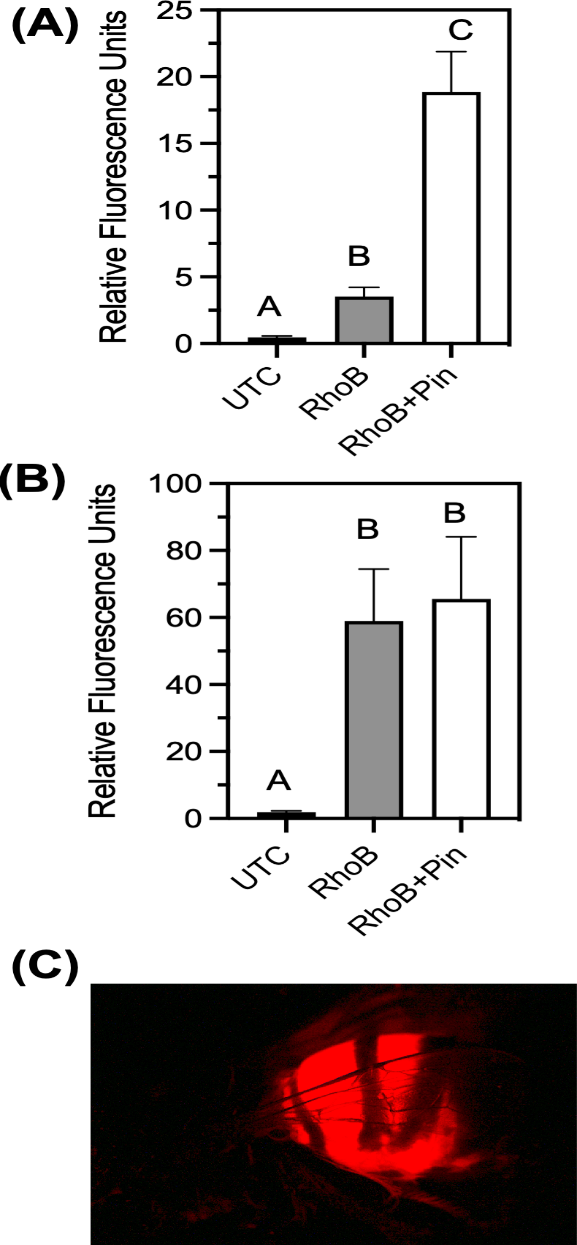
Additional file 1: Fig S2. Validation of treatment solution feeding method using Rhodamine B.** Bars represent mean (n=30) relative fluorescence units of bee (**A)** and nectar samples (**B)**. Comparisons were made between bees receiving sucrose alone (UTC), sucrose plus rhodamine (RhoB) and sucrose plus rhodamine and Pinacidil (RhoB + Pin). (**A**) The fluorescence of bee abdomens was compared 24 hours after application of treatment solution. (**B**) Comparison of fluorescence of samples of freshly stored nectar. For A-B, bars not labeled by the same letter represents statistical significance at P<0.05 as determined by one-way ANOVA with Tukey’s posttest. Error bars represent SEM. (**C**) Representative image below figure shows fluorescence of bee exposed to Rhodamine B + Pinacidil.

**
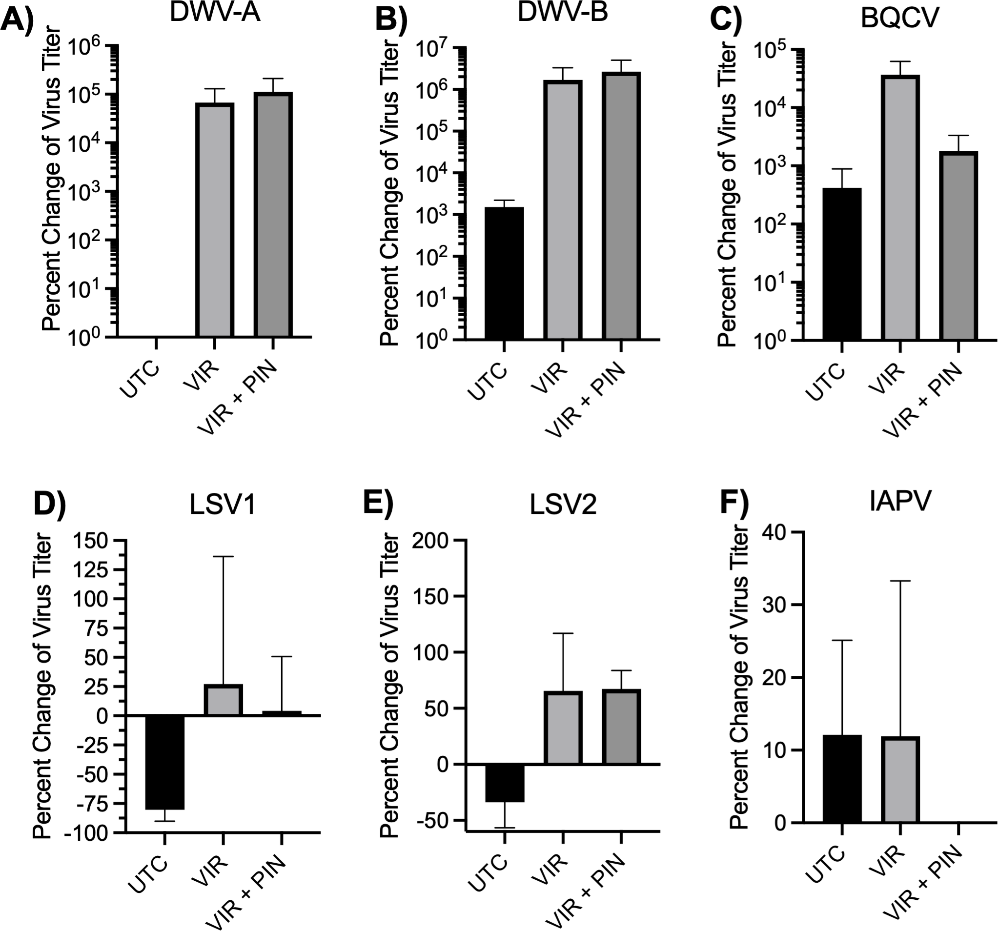
Additional file 1: Fig. S3. The effect of pinacidil exposure on virus infection in nurse bees in inoculated colonies.** Viruses screened include DWV-A (**A**), DWV-B (**B**), BQCV (**C**), LSV1 (**D**), LSV2 (**E**), and IAPV (**F**). Bars represent average (n=10 colonies) percent change in virus titer among emerging bees between initial and post-treatment timepoints and error bars represent SEM. Treatment groups include untreated (UTC), virus only (VIR), and 2 mM pinacidil + virus (VIR + PIN).
